# *Jatropha curcas*, a biofuel crop: Functional genomics for understanding metabolic pathways and genetic improvement

**DOI:** 10.1002/biot.201300231

**Published:** 2013-10-02

**Authors:** Fatemeh Maghuly, Margit Laimer

**Affiliations:** Plant Biotechnology Unit, Department of Biotechnology, BOKU-VIBT, University of Natural Resources and Life SciencesVienna, Austria

**Keywords:** Biofuel, Breeding, Domestication, Purging nut

## Abstract

*Jatropha curcas* is currently attracting much attention as an oilseed crop for biofuel, as *Jatropha* can grow under climate and soil conditions that are unsuitable for food production. However, little is known about *Jatropha*, and there are a number of challenges to be overcome. In fact, *Jatropha* has not really been domesticated; most of the *Jatropha* accessions are toxic, which renders the seedcake unsuitable for use as animal feed. The seeds of *Jatropha* contain high levels of polyunsaturated fatty acids, which negatively impact the biofuel quality. Fruiting of *Jatropha* is fairly continuous, thus increasing costs of harvesting. Therefore, before starting any improvement program using conventional or molecular breeding techniques, understanding gene function and the genome scale of *Jatropha* are prerequisites. This review presents currently available and relevant information on the latest technologies (genomics, transcriptomics, proteomics and metabolomics) to decipher important metabolic pathways within *Jatropha*, such as oil and toxin synthesis. Further, it discusses future directions for biotechnological approaches in *Jatropha* breeding and improvement.

## 1 Introduction

The species *Jatropha*, family *Euphorbiaceae*, native to Central America, was spread by Portuguese seafarers via Cabo Verde and Guinea Bissau to other countries in Africa and Asia [1–3]. Today, this subtropical plant is widespread in different agricultural systems as hedges, wind protection systems, erosion barriers or as a source of firewood [2, 3]. In addition, different parts of *Jatropha* contain a range of interesting metabolites and medicinal components [4, 5], which have long been used as raw material for lamp oil, soap production, paints, lubricating oils, and for medical applications [5–7]. *J. curcas* has a relatively small genome (2C DNA content of 0.850 ± 0.006 pg or 1C DNA content of 0.416 × 10^9^ bp) organized in 22 chromosomes [8], which makes it an attractive candidate for genome sequencing and genomic analyses. *J. curcas*'s close taxonomic distance to important *Euphorbiaceae* species, such as *Ricinus* or *Manihot*, allows the comparison of diverging orthologous to partial genomes of these species.

This stem-succulent tree produces seeds containing 30–45% toxic oil, with a high percentage of monounsaturated oleic and polyunsaturated linoleic acid [9–11]. The press cake from seeds provides organic manure, and is rich in protein (60–63%) compared to soybean (45%). The press cake could be an ideal protein source with a high content of essential amino acids even higher (except lysine) than the Food and Agriculture Organization reference protein [12]. However, seeds of *J. curcas* contain a range of toxins and anti-nutritional compounds, which render the seedcake and oil unsuitable for use as animal feed or for human consumption [13]. In addition, plantations are planted with seeds from undomesticated plants that represent anything but uniform genotypes, and due to inefficient management, it is not possible to reliably predict yield. Likewise, a lack of knowledge of the reproductive biology, the quantitative genetic variations, the interaction of genotype and environment, and gene expression patterns under variable environmental conditions make it difficult to predict yields and toxin levels.

Access to the domestication of *Jatropha* requires a holistic approach, in order to retain valuable genetic resources. Developing *Jatropha* cultivars for a wide variety of applications (e.g. biofuel production, reforestation, medical applications, soap production) and with a number of special features (growth form, high yield, adaptation to different climatic conditions, high oil content, lower toxin levels, reduced allergenicity and pathogen resistance) will allow for an economic use of the plant. To make an informed selection of plant material and to assign functions to selected gene products, a deep understanding of the physiology of *Jatropha* for practical applications is required. Therefore, this review focuses on: (i) high-throughput technologies for gene identification, (ii) functional genomics analysis of economic traits such as oil and toxin, (iii) advanced breeding technologies, and (iv) future directions of *Jatropha* improvement.

## 2 High-throughput technologies for gene identification in *J. curcas*

Over the past 10 years, high-throughput and cost-effective sequencing methods have been developed that can accelerate the process of breeding [14, 15]. Since 2010, several sequences from the cDNA library of *J. curcas* seed have been published [16–20]. Sequences of 2200 clones containing 931 unigenes [16] and 12 084 expressed sequence tags (ESTs) with 2258 contigs, 4751 singletons and 7009 unigenes have been identified, encoding diverse biological functions including oil and toxin biosynthesis [17]. Costa et al. [18] generated 13 249 ESTs and, subsequently, Natarajan and Parani [21] generated 17 457 assembled transcripts and 54 002 singletons with an average length of 916 bp. BLASTX (http://blast.ncbi.nlm.nih.gov/) analyses showed that 2589 of these transcripts are full length and the Kyoto Encyclopedia of Genes and Genomes (http://www.genome.jp/kegg/) automatic annotation server showed that 2320 transcripts are involved in major biochemical pathways, including oil biosynthesis [19].

The transcriptome of developing *J. curcas* seeds produced 195 692 sequences (46 Mbp) of raw sequence data, assembled to 12 419 contigs and 17 333 singletons [20]. A combination of the conventional Sanger and next generation sequencing methods of *J. curcas* produced, in total, 285 858 490 bp consisting of 120 586 contigs and 29 831 singlets and 40 929 complete and partial protein-encoding genes [21]. The sequences and annotations were later upgraded by the addition of new data, combining the 1 025 000 reads from the conventional Sanger method and 2 312 828 reads of the next generation sequencing method [22]. Based on an *ab initio* predicted analysis, 30 203 potential protein-encoding genes were identified, of which 2402 genes were ascribed to 19 metabolic pathways [22].

## 3 Functional genomics analysis of economically important traits in *J. curcas*

Functional genomics seeks to determine gene function through the correlation of genes and their products. These reverse genetic approaches are followed by simultaneous profiling of gene expression products termed transcriptomics, proteomics and metabolomics (http://www.noble.org/plantbio/sumner/functional-genomics/). In addition, with the advance of genome sequencing in *J. curcas*, the expression of genes involved in the biosynthetic pathways have become more feasible, which should be beneficial for selective breeding of *J. curcas*, especially for oil quality, yield and low toxins [3]. The identification of relevant loci will facilitate the breeding of new dual-purpose edible seeds to be used as a source of oil and animal feed [23]. Here we focus on two major economically important traits, fatty acid (FA) and toxin biosynthesis in *J. curcas.*

### 3.1 FA metabolism of *J. curcas*

**Figure 1:**
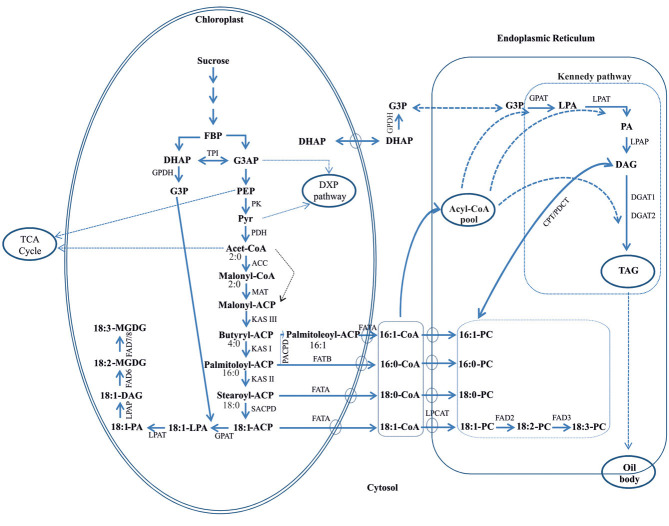
The biosynthesis pathway of the FAs in plants. ACC, acetyl-CoA carboxylase; ACP, acyl carrier protein; CoA, coenzyme-A; CPT, CDP-choline:diacylglycerol cholinephosphotransferase; DAG, diacylglycerol synthase; DGAT, diacylglycerol acyltransferase (DGAT1;DGAT2); DHAP, dihydroxyacetone phosphate; FAD2, oleoyl-phosphatidylcholine Δ-12 desaturase; FAD3, linoleoyl-phosphatidylcholine ω-3 desaturase; FAD6, Δ-12 desaturase; FAD7/8, ω-3 desaturase; FATA, stearoyl-ACP thioesterase A ; FATB, Palmitoyl-ACP thioesterase; FBP, fructose bisphosphate; GA3P, glyceraldehyde-3-phosphate; G3P, glycerol-3-phosphate; GPAT, glycerol-3-phosphate O acyltransferase; GPDH, glycerol-3-phosphate dehydrogenase; LPA, lysophosphatidic acid; LPAT, lyso PA acyltransferase); LPCAT, lyso PC acyltransferase; LPAP, lyso PA phosphatase; MAT, ACP-S-malonyl transferase; MGDG, galactolipid monogalactosyldiacylglycerol; KAS, beta-ketoacyl-ACP synthase (KAS I; KASII; KAS III); PA, phosphatidic acid; PACPD, palmitoleoyl-ACP Δ9-desaturase; PC, phosphatidylcholine; PDCT, phosphatidylcholine:diacylglycerol cholinephosphotransferase; PDH, pyruvate dehydrogenase; PEP, phosphoenolpyruvate; PK, pyruvate kinase; Pyr; pyruvate; SACPD, stearoyl-ACP Δ9-desaturase; TAG, triacylglycerol; TPI, triose-phosphate isomerase.

Plant lipids are synthesized as triacylglycerols (TAGs) via a complex series of pathways in which many FA biosynthetic enzymes are involved (Fig. 1). Although the biosynthesis of FAs is well understood, little is known about the regulation, amount, or type of FAs produced in different tissues or organs such as seeds [24]. The major FAs in plant oils are palmitic (16:0), stearic (18:0), oleic (18:1), linoleic (18:2) and linolenic acids (18:3). Palmitic and stearic acids are saturated, oleic acid is monounsaturated, and linoleic and oleic acids are polyunsaturated FAs [25]. The ability of biodiesel to meet the special criteria is largely determined by its FA composition. In addition, cold flow, cloud point properties, kinetic viscosity, oxidative stability and the cetane number (CN), an important parameter to determine the ignition quality for biodiesel, are influenced by the FA composition [20, 26]. Biodiesel containing high levels of monosaturated FAs is preferable, while high levels of polyunsaturated FAs decrease biodiesel stability, increase the oxidative stability and affect the CN [25]. Thus, identification of processes to manipulate and to modify the FA composition of candidate resources would be highly desirable [27]. The saturated FA content of *J. curcas* oil includes 14.1–15.3% palmitic acid, 3.7–9.8% stearic acid, 34.3–45.8% oleic acid and 29.0–44.2% linoleic acid [9]. Therefore, to improve *Jatropha* biodiesel qualities, higher oleic acid (>70%) and lower saturated FA (<10%) would be required, which can be achieved by altering the FA composition in *Jatropha* seeds ([28], see Section 4.2).

Transcriptomic analyses of genes involved in FA biosynthesis can provide fundamental molecular understanding of synthesis and storage of lipids and proteins in *Jatropha* seeds. The expression levels of different key genes involved in FA biosynthesis in developing seeds (14–45 days) after pollination (AP) showed that most genes were upregulated between 29 and 41 days AP and the expression of most oil-body protein genes increased from 35 days AP [27]. Interestingly, electron microscopy showed that the oil-body formation appeared at 28 days AP, was actively developed by 42 days AP and reached a maximum number and size after 56 days AP [24]. Annarao et al. [29] found that lipid synthesis initiated 3 weeks after fertilization and TAGs were synthesized actively between developmental stages IV and VII.

More recently, Gu et al. [24] showed that genes with similar functions were expressed differentially during endosperm development, and the majority of FA and lipid biosynthetic genes are highly consistent with the development of oil bodies and endosperm in *Jatropha* seeds.

Temporal expression profiles of 21 lipid genes involved in FA and TAG synthesis pathways revealed that the expression of 17 genes was increased in developing *Jatropha* seeds compared to leaves [30]. Only two diacylglycerol acyltransferase genes (DGAT1 and 2), representing rate-limiting enzymes in plant lipid accumulation, were specifically associated with the biosynthesis of TAG [30]. In accordance, Jiang et al. [27] found that the expression of β-ketoacyl-acyl carrier protein (ACP) synthase I (KAS I) gene increased before that of KAS II and KAS III, catalyzing the initial step of FA biosynthesis. In contrast, no comparable expression of genes involved in TAG synthesis was found in comparison to other oilseed plants [27, 31]. Studies of the expression of *Jatropha* KAS III in different tissues revealed the highest expression in roots and in developing seeds, which even increased with time [32, 33].

Costa et al. [18] identified most genes involved in FA biosynthesis, except KAS III and hydroxyacyl-ACP dehydrase. They also found ESTs coding for enzymes that produce oleic and stearic [stearoyl-ACP thioesterase A (FAtA)], linoleic and palmitic [FAtA and palmitoyl-ACP thioesterase (FAtB)] acids and oleate desaturase (FAD2), which catalyze oleoyl-ACP (oleic) to linoleoyl-ACP (linoleic). King et al. [20] reported transcripts corresponding to the cytosolic glycolysis pathway, the plastidial glycolytic and Kennedy pathway, which is the most important route to TAG biosynthesis (Fig. 1).

Despite the progress achieved in genomic and transcriptomic studies, especially of FA biosynthesis in *J. curcas*, there are only few protein-based and proteomic studies available [33]. Staubmann et al. [34] extracted a new lipase that has potential use in lipid modification from the seeds of *J. curcas.* Lipase activity was absent in dormant seeds, but activity increased during germination and reached a maximum 4 days after germination. Liu et al. [35] compared the protein profiles of embryo and endosperm of seeds using two-dimensional gel electrophoresis, which yielded 380 and 533 major protein spots, respectively. They found 27 spots for proteins participating in the tricarboxylic acid cycle. Most of the proteins in the endosperm were catabolism-related enzymes, i.e. providing nutrients for germination, while most of the proteins in the embryo were related to anabolism, i.e. utilizing the nutrition from the endosperm.

To understand the mobilization, biochemistry and metabolisms involved in the synthesis and breakdown of storage oil in *Jatropha* seeds, Yang et al. [36] extracted protein from the endosperm of seeds germinated for 0, 24, 48 and 60 h. Popluechai et al. [37] isolated and characterized three single-copy oleosins (*JcOle1*, *JcOle2*, *JcOle3*), which control the size of oil bodies and contain a proline knot domain. They also analyzed the transcript and protein levels of these oleosins. Investigations on the C_3_/C_4_ photosynthesis in *J. curcas* showed that phosphoenol pyruvate carboxylase, known to contribute to fatty acid metabolism and photosynthesis, was unique in that it contained only six exons, whereas almost all plants contain 10 exons [33].

Metabolomics studies on lipid profiling of *J. curcas* seeds collected at various developmental stages (I–VII) using proton nuclear magnetic resonance spectroscopy, revealed the presence of free FAs, methyl esters of fatty acids and TAG, together with small quantities of sterols [29].

### 3.2 Toxin biosynthesis in *J. curcas*

The identification of genes responsible for the biosynthesis of anti-nutritional compounds is important for the improvement of *Jatropha* seeds as valuable by-product for livestock feed [38]. The toxicity of *J. curcas* seed has been mainly attributed to the presence of diterpenes (phorbol esters; PEs), ribosome-inactivating proteins (RIP; curcin), saponins, trypsin inhibitor, protease inhibitors, curcain, jatrophidin, phytates, alkaloids, lectins, lignans, tannins, latex and cyclic peptides [5].

#### 3.2.1 Diterpenes

**Figure 2:**
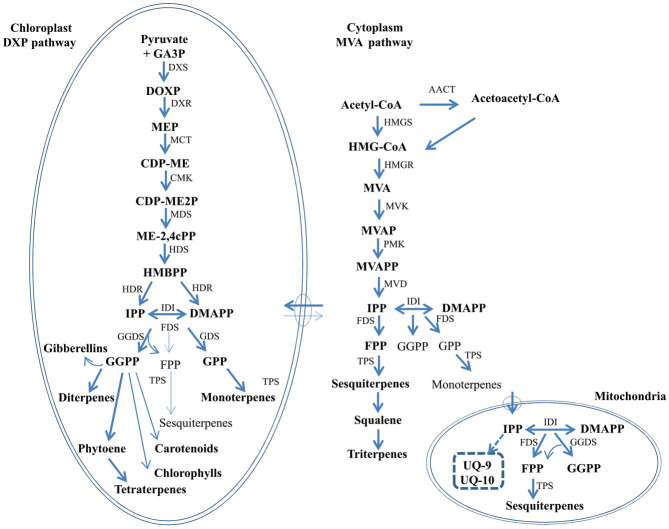
The biosynthesis pathway of the major terpenoids in plants. AACT, acetoacetyl-CoA thiolase; AcAc-CoA, acetoacetyl-CoA; CDP-ME, 4-(cytidine 50-diphospho)-2-C-methyl-d-erythritol; CDP-ME2P, 4-(cytidine 50-diphospho)-2-C-methyl-d-erythritol phosphate; CMK, CDP-ME kinase; DMAPP, dimethylallyl diphosphate; DOXP, 1-deoxy-d-xylulose 5-phosphate; DXR, DOXP reductoisomerase; DXS, DOXP synthase; FDS, farnesyl diphosphate synthase; FPP, farnesyl diphosphate; GA3P, glyceraldehyde-3-phosphate; GDS, geranyl diphosphate synthase; GGDS, geranylgeranyl diphosphate synthase; GGPP, geranylgeranyl diphosphate; GPP, geranyl diphosphate; HDR, (E)-4-hydroxy-3-methylbut-2-enyl diphosphate reductase; HDS, (E)-4-hydroxy-3-methylbut-2-enyl diphosphate synthase; HMBPP, (E)-4-hydroxy-3-methylbut-2-enyl diphosphate; HMG-CoA, 3-hydroxy-3-methylglutaryl-CoA; HMGR, HMG-CoA reductase; HMGS, HMG-CoA synthase; IDI, isopentenyl diphosphate isomerase; IPP, isopentenyl diphosphate; MCT, 2-C-methyl-d-erythritol 4-phosphate cytidylyltransferase; MDS, 2-C-methyl-d-erythritol 2,4 cyclodiphosphate synthase; ME-2,4cPP, 2-C-methyl-d-erythritol 2,4-cyclodiphosphate; MEP, 2-C-methyl-d-erythritol 4-phosphate; MVA, mevalonic acid; MVAP, mevalonate-5-phosphate; MVAPP, mevalonate-5-pyrophosphate; MVD, mevalonate diphosphate decarboxylase; MVK, mevalonate kinase; PMK, phosphomevalonate kinase; TPS, terpene synthase.

Diterpenes (C_20_H_32_) are organic compounds composed of four isoprene units, which derive from geranylgeranyl diphosphate (GGPP) (Fig. 2), and represent the most widely distributed terpenes in the plant kingdom [39]. Although known for their antimicrobial and anti-inflammatory properties, diterpenes also show cytotoxic effects on mammalian cells [39]. *J. curcas* contains high amounts of diterpenes such as tigliane, casbene, daphnane, lathyrane, jatrophane and podocarpane [5]. So far, only the gene for GGPP synthase (*GGPPS*) has been reported to be involved in the biosynthesis of PEs in *J. curcas.* This was confirmed by functional analyses of Jc-GGPPS in GGPPS-deficient mutant plasmids, which showed the influence of *Jc-GGPPS* on carotenoid biosynthesis and as general precursor for diterpene biosynthesis [40]. Therefore, any manipulation of this gene may alter the PE content of *Jatropha* seeds [33].

Tigliane, a tetracycline diterpene, the fundamental alcohol moiety in PE (phorbol-12-myristate-13-acetate), contains four rings (A–D) [12, 41]. Due to their low abundance, extreme instability and susceptibility to oxidation, hydrolysis and transesterification, so far only six different compounds have been isolated from *J. curcas*, and named *Jatropha* factors C1 to C6 ([41], Supporting information, Fig. S1]. All contain the same intramolecular diesters of the same diterpene, namely, 12-deoxy-16-hydroxyphorbol. PEs are lipophilic, and so present mainly in the oil; they are also heat-stable [42]. PEs also activate protein kinase C (PKC), a phosphorylated regulatory protein that activates other metabolic pathways, such as ion channels or gene transcriptions [42, 43]. Normally, PKC is activated by diacylglycerol, which has a short biological half-life in the cell. However, PE acting as analog of diacylglycerol activates PKC more strongly and for prolonged time periods, which can lead to a number of biological processes [42, 43]. Isolated PEs induce a wide range of biochemical and cellular effects such as inflammation, tumor promotion, and cell proliferation [12]. PEs have been tested as antiparasitic drugs and for their tumor-inducing effects demonstrated in mice [39, 44].

Studies analyzing spatial distribution, location and tissue of origin of PEs in *Jatropha* seeds could help us understand the pathways involved. The seed coat in *Euphorbiaceae* contains both tegmen and testa that originate from outer and inner integument of the maternal ovary [45], which led Sujatha et al. [46] to suggest that the production of PE is controlled maternally. Recently, He et al. [23] showed that PEs were absent from the testa; small amounts of PEs (<30 μg/g) were observed in the embryo, and high amounts (347 μg/g) in the endosperm, while the highest concentration was found in the tegmen (2755 μg/g).

Non-toxic varieties from Mexico were reported to contain negligible concentrations (0.27 × 10^3^ μg/ml), while toxic varieties contained 2.49 × 10^3^ μg/ml PEs [43]. However, non-toxic selections from Mexico contained only 5% of PEs, but still 50% of curcin, and about 25% more trypsin inhibitor and 50% more saponins [47]. Francis et al. [48] introduced a non-toxic variety, based on PE content, that produces similar or better seed yield per plant, seed oil content, and FA composition than toxic varieties. The use of either naturally occurring or novel cultivars with reduced toxin contents would abate the costs associated with conversion of seed cake to animal feed, generating additional revenues from *Jatropha* [23].

An isoprenoid biosynthesis gene and its protein, 3-hydroxy-3-methylglutaryl coenzyme A reductase, which catalyzes the first committed step in mevalonic acid synthesis and leads to carotenoids and PEs synthesis, were isolated from *Jatropha* and characterized [49]. The full-length cDNA consisted of a 1950-bp open reading frame encoding 584 amino acids, and contained two transmembrane and one catalytic domain that had a high identity to other plants [49]. Similarly, the cloning and characterization of a full-length cDNA encoding GGPPS (*JcGGPPS*) from *J. curcas* have been reported [40]. This comprised 1414 bp, with an 1110-bp open reading frame encoding a 370-amino acid polypeptide. Bioinformatics analyses revealed that the JcGGPPS is a member of the polyprenyltransferases with two highly conserved aspartate-rich motifs, and high homology to other plants [40]. However, further characterization of the *JcGGPPS* gene is needed to gain a better understanding of the regulation of PE biosynthesis in *J. curcas*, and to achieve decreasing toxin levels using biotechnology [40].

#### 3.2.2 Ribosome-inactivating proteins

One class of toxic proteins found in many plants, fungi and bacteria are the so-called RIPs, which play an important role by inactivating ribosomes and thus inhibiting protein synthesis [50, 51]. Some studies report that RIPs may be involved in the plant defense response [52–54]. Although RIPs have been classified into three groups, based on their structure and functions, type I RIPs are most important in *J. curcas.*

Type I RIPs consist of a single polypeptide chain with enzymatic activity, and have a molecular mass of about 23–32 kDa and an alkaline isoelectric point of pH 8–10 [50]. Type I RIPs, including curcin from *J. curcas*, are relatively non-toxic to cells and animals, but can act as antiviral and antitumor proteins [5, 52, 55]. Their inactivating mechanism relates to *N*-glycosidase activity, which cleaves the *N*-glycosidic bond of adenine A4234 of 28S rRNA. This makes ribosomes unable to bind elongation factors 1 or 2, and consequently arrests protein synthesis [5, 50, 55]. Lin et al. [50] observed an antitumor effect of curcin on SGC-7901, Sp2/0 and human hepatoma cells, although curcin had no effect on HeLa and normal cells. Curcin contains one cysteine residue, which might be suitable for forming a disulfide bond with an activated antibody as a chemotherapeutic treatment for cancer [5, 55]. A disulfide linkage is usually thought to be essential for maximum cytotoxicity, but it is also necessary to chemically modify the antibody and the RIP to produce the disulfide bond [50].

Curcin was isolated, cloned and sequenced [50, 54, 56]. Three contigs encoding amino acid sequences of curcin-like proteins were identified as highly similar to curcin [21]. King et al. [20] found that curcin represented 0.7% of the transcriptome, while no transcripts for type II RIPs were found in seeds of *J. curcas.*

Preliminary proteomics by Costa et al. [18] identified five curcin isoforms in the developing and germinating seed libraries. Curcin-L was expressed and activated in the leaves of *J. curcas* by treatments with abscisic acid, salicylic acid, polyethylene glycol, extreme temperatures and ultraviolet light [54]. Curcin 2 was isolated from leaves that had been subjected to drought, temperature, and fungal infection stresses [57], suggesting a role in plant defense. In addition, these responses showed the ability of *J. curcas* to adapt to various environmental conditions. Comparison of the proteins from toxic and non-toxic *Jatropha* seeds showed that both contain a single protein of 28 kDa. In addition, curcin was detected in the tegmen and endosperm, but not in the embryo [23].

## 4 Breeding technologies for *Jatropha*

The ultimate breeding objectives are high oil yield and reduced toxicity, without impairing the natural pathogen resistance, and ensuring the protection of animals. Selection and multiplication of superior germplasm for quality planting material is now the prime aim for achieving domestication and improvement in productivity of the species under adverse climate conditions [58]. However, using conventional breeding, the process from hybridization to cultivar release can span decades. Achten et al. [38] estimate that, because *J. curcas* is a semi-wild plant, it will require a minimum 15 years of conventional breeding before *Jatropha* reaches a level of domestication. This period could be shortened if plant tissue culture and improvement through transgenesis were used [28, 59–62].

However, a high level of genetic variation is of crucial importance for breeding programs [7]. The lack of knowledge about the genetic constitution of the plant material limits the success of breeding programs and makes it harder to exploit the full potential of *Jatropha.* Knowledge about the degree of genetic diversity among naturally occurring populations within and outside of the accepted “Center of Origin” in Central and South America allows a targeted search for interesting backgrounds and to develop appropriate breeding strategies [38]. Traditional methods using morphological characteristics to determine genetic diversity or proximity of different provenances of *J. curcas* were only of limited success, primarily because of environmental influences on otherwise very stable hereditary characteristics such as 1000 grain weight, protein and oil content of seeds [1]. Trabucco et al. [63] carried out a novel two-step approach based on knowledge from biogeography and population biology with available *Jatropha* field data, showing that climate changes, e.g. in annual average temperature, minimum temperature, annual precipitation and precipitation seasonality, are most significantly affecting yield responses. Higher levels of chemical, floral and molecular variability were found in Mexican than in South American accessions of *Jatropha* [64]. Over the past few years, molecular markers have been used to genetically characterize the *J. curcas* germplasm, yielding contrasting results from high to rather low genetic diversity, which might be explained either by the number of accessions or the techniques used [13, 38, 60].

Additional studies are required to shed light on DNA polymorphism levels within geographical ecotypes; these levels are important cornerstones for genetic conservation and selection programs in this species. Further, due to the low number of cloned genes and its largely uncharacterized genome, *J. curcas* is a species requiring major research initiatives in agronomy and biotechnology with the aim of breeding new genetically improved varieties [65]. For instance, the adoption of transgenesis for the improvement of biofuel crops, including *J. curcas*, has been recently recommended [59], while the exploitation of interspecific crosses among closely related *Jatropha* species was postulated as a strategy for the development of new varieties [66]. However, for domestication, selection of promising individuals, germplasm collection and interspecific hybridization are necessary. Reddy et al. [67] produced crosses between *Ricinus communis* and *J. curcas* and five related species. Based on pollen germination, *J. curcas* showed a closer relationship to *J. gossypiifolia* and *J. glandulifera.* Later experiments involving interspecific hybridization between *J. curcas* and related species showed successful progenesis [66, 68, 69]. A cross between *J. curcas* and *J. integrrima* resulted in successful seed production and allowed backcrossing with *J. curcas* [3].

Nevertheless, the lack of high genetic variability in *J. curcas* hampers selective breeding [70], which calls for other strategies to increase the genetic diversity through chemical/physical mutations or intra/interspecific crossing programs [13].

### 4.1 Quantitative trait locus analysis

Since the breeding of *J. curcas* is still in its early stages, and there is a lack of pedigree with high phenotypic segregation, the development of inbred lines will be a good strategy for producing segregating crosses [3]. A first-generation linkage map was generated by Wang et al. [71] using a mapping panel containing two backcross populations between *J. curcas* and *J. integerrima.* The same backcross population containing 286 individuals was used to identify 18 quantitative trait loci (QTLs) underlying the oil traits [72]. Single nucleotide polymorphisms (SNPs) of three oleosin genes [37] were mapped onto the linkage map, identifying three expression QTL (qC18:1-1, qOilC-4 and qOleIII-5), which control oleic acid, oil content and oleic gene expression, respectively [72]. More recently, Sun et al. [73], using the same backcross population, produced 28 QTL for 11 growth and seed traits. Two QTLs controlling seed yield were conferred by the alleles from *J. curcas*, while 5 QTLs controlled plant height, branch number, female flower number and fruit number conferred by the alleles from *J. integerrima* [73].

Most genes involved in TAG biosynthesis in *Jatropha* appear as a single gene for every enzyme isoform, and, interestingly, no obvious gene duplication was found [21]. Therefore, some of the QTLs related to oil quality have been mapped to these genes [16, 71].

It is suggested that *Jatropha* cDNA corresponding to geranyl diphosphate synthase (GPPS) could be used as a QTL related to PEs biosynthesis [16]. However, it is known that GPPS is also involved in the synthesis of cytokinins, which makes GPPS unsuitable as a QTL marker [3]. Marker-assisted selection is effective only when the markers are tightly linked to the gene of interest and a single trait. Therefore, more molecular markers (especially SNPs) should be identified and more ESTs or gene sequences mapped to find QTLs for important traits. This will allow genome-wide association studies to be conducted and provide a better understanding of the causative mutations of phenotypic variations [71]. Reverse genetics for haplotyping and SNP discovery like EcoTILLING [70] and RAD sequencing can make significant contributions to trait correlation, especially for complex trait controlled by multiple genes or QTLs.

### 4.2 Genetic modification of *Jatropha*

Genetic transformation for plant improvement [13] relies on several transformation methods; so far *Agrobacterium* or particle bombardment for genetically modifying oil and toxin biosynthesis in *Jatropha* have been reported [28, 60–62].

Increasing oil yield and/or improving the FA composition are the obvious approaches for developing a premium sustainable bioenergy crop [15, 25, 28]. In addition, genes involved in the biosynthesis pathway of TAGs are of great interest to improve oil accumulation in *Jatropha* seeds by genetic modification ([15], Fig. 1). Due to the commercial importance of the oil, genes of enzymes involved in the FA metabolism have also been cloned. Full-length cDNA of *JcFATB*, which is located in the chloroplast, has been isolated from *J. curcas*, and has been shown to encode a transit peptide that is processed in multiple steps and involved in the termination of carbon chain elongation [74, 75]. The *JcFATB1* single copy gene is active in all tissues, mainly in roots, and is increasingly expressed during seed development [74]. Overexpression of the *JcFATB1* cDNA in *Arabidopsis* resulted in increased levels of saturated FAs such as palmitate, while unsaturated FA levels were decreased [74]. Similarly, a full-length cDNA encoding stearoyl-ACP Δ9-desaturase was obtained from developing seeds of *J. curcas* [76], and functionally expressed in *E. coli.* stearoyl-ACP Δ9-desaturase plays an important role in FA biosynthesis and also determines the ratio of saturated and unsaturated FAs [77]. The gene is member of a small gene family and is upregulated in developing fruits [76]. In addition, overexpression (under the CaMV 35 S promoter in *Arabidopsis*) of KASII of *J. curcas*, which is responsible for carbon chain elongation in the FA biosynthesis, led to a changed FA composition in seeds and leaves by decreasing 16-carbon FAs and increasing 18-carbon FAs [78].

The first transformation using the inducible Cre/Lox system to obtain marker-free transgenics was reported to improve agronomical traits and seed quality [25]. One of three putative *JcFAD2s* genes mediating the conversion of oleic to linoleic acids was downregulated by RNA interference technology [25]. Downregulation of *JcFAD2-1* increased the oleic acid content (>75%), while reducing polyunsaturated FAs (<3%) in *Jatropha.* The CN after these changes in the FAs profile was estimated to be as high as 60.2, which corresponds to the requirements for diesel in Europe. Four subunit genes of heteromeric acetyl-CoA carboxylase, which is involved in the essential first step in the biosynthesis of long-chain FAs, were isolated from *Jatropha* [79]. The single-copy *accA*, *accB1* and *accC* are nuclear genes and the *accD* is a plastid gene, and all are expressed temporally and spatially in leaves and endosperm [79]. Combining *KASII* overexpression with *FAD2-1* downregulation using RNA interference technology in *Jatropha* will increase both oleic acid and unsaturated FAs contents, which in turn also affects the oxidative stability of *Jatropha* biofuel [28].

### 4.3 Induced mutagenesis

Plant breeding is defined as identifying and selecting desirable genetic variations of useful traits for crop improvement. Often, however, when desired variation is lacking [80], physical and chemical mutagenesis, which cause localized sequence changes, offer additional approaches to increase the genetic variability or to change expression levels or patterns of genes [81]. Another type of specific gene manipulation for replacing an existing sequence with a designed one is target gene replacement by zinc-finger nuclease [82].

Treating *J. curcas* seeds with gamma radiation at 10–40 kR induced an increased germination rate, but decreased seedling height [83]. Some morphological changes were detected in M2 plants, such as tricotyledonous seedlings, early branching stems, early flowering, and plants with entire leaf margins in some branches [83]. Studies of Dhakshanamoorthy et al. [84, 85] showed that the effects of gamma radiation (5–25 kR) were greater than those of ethyl methanesulfonate (EMS) (1–4%) on seed germination, flowering, fruit and seed traits. In addition, low doses of gamma radiation (5 kR) caused early flowering, higher fruit and seed yield. Higher doses, however, led to a reduction in all parameters. Further, 1% EMS had a stimulatory effect on germination percentage, growth and fruit traits, while 4% EMS showed an inhibitory effect on all selected parameters.

Treating dry seeds with single or combined treatments of gamma radiation (6, 12 or 18 kR) and colchicine (0.25, 0.5 or 1.0%) showed that seed germination was affected by gamma radiation of 12 and 18 kR and 0.25% colchicine, while germination time was significantly delayed by higher concentrations of colchicine [86]. Different types of leaf size reductions and abnormity were observed in most treatments. However, in the second year, individual plants showed a wide variability in reaction to different treatment combinations [86].

To our knowledge, so far no genetic analyses have been carried out on mutagenized *Jatropha* populations. This was recently achieved with TILLING (targeting induced local lesions in genomes), which is a reverse genetics method that can be used for the characterization of in vivo functions of genes. It combines random mutagenesis with high-throughput mutation discovery, provides a spectrum of stable point-alleles and is broadly applicable across most species [70, 87].

## 5 Future directions of *Jatropha* improvement

Genome sequencing and systems biology have revolutionized plant functional genomics. Once expression has been altered, mRNA, protein and/or metabolite levels are quantified through various profiling approaches. Knowledge about the pattern of gene expression in plant tissues under variable culture conditions will help to increase production efficiency. To understand processes such as maturation and seed quality, determined by the production of oil, FAs or toxins, transcriptomics must be performed on successive stages of developing seeds [24]. The identification and characterization of the spatial and temporal expression of genes that are economically important for FA biosynthesis will help us understand their regulation. The information on gene expression levels and patterns could provide the necessary data for breeding and genetic engineering to increase oil content or optimize FA composition in *Jatropha* seeds [24].

Furthermore, proteomic approaches generate great insight into the plant systems biology in general and can explore the metabolic pathways of *Jatropha* in particular [14, 88]. Knowledge of the protein content and distribution patterns in developing seeds as the most interesting tissue for oil production deserves special attention, and will provide details of the regulatory mechanisms in *Jatropha.* Therefore, there is a need for essential technologies to enhance the detection of low abundant proteins, as well as protein annotation such as targeted and non-targeted protein analyses [14].

Contrary to proteomics, for which the analysis can start from genomic sequences, for metabolomics there is no initiation reference [4]. Metabolomics [88] are a challenging endeavor since no single analytical approach addresses all the different chemical structures, and many different techniques are needed to cover different compound classes with their diverse chemical properties [14]. Although 164 compounds [4] and some important metabolic pathways in *Jatropha* have been recognized, our understanding about complex pathways and how they are regulated under different stress conditions are far from being complete.

**Figure 3:**
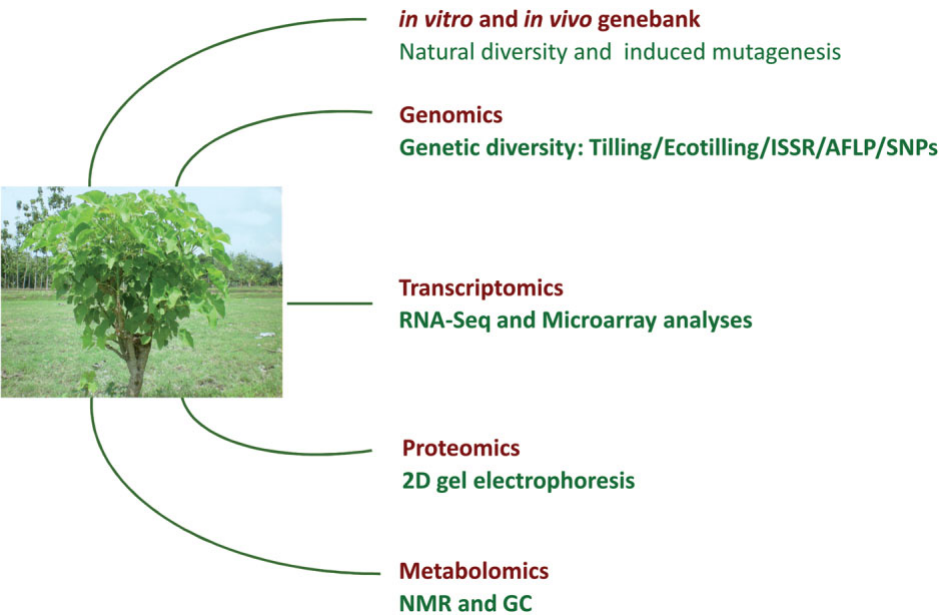
Functional genomic analyses were applied by the Plant Biotechnology Unit to determine how and when oil and toxins are produced in developing seeds and to identify possible impacts on the strategies for *Jatropha* genetic improvement.

Therefore, investigations of functional genomics for important metabolic pathways will support the understanding and the improvement of *J. curcas* as a source of biofuel, as shown in Fig. 3. Without such deep understanding, *J. curcas* cannot be improved to meet the criteria required for a valuable feedstock [33].

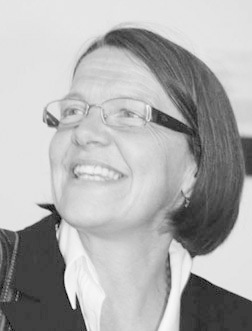

Professor **Margit Laimer** is the leader of the Plant Biotechnology Unit (PBU), Department of Biotechnology, VIBT-BOKU, University of Natural Resources and Life Sciences, Vienna, Austria. She is an expert in plant biotechnology. Originally trained in botany, with specialization in plant tissue culture, she holds two habilitations in the related fields plant biotechnology and plant virology. Her fields of research are plant health and plants for human health.

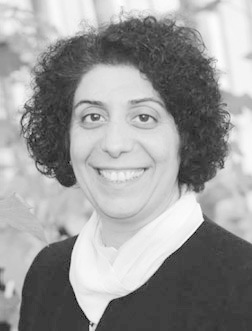

Dr. **Fatemeh Maghuly** was originally trained in botany, with specialization in population genetics and molecular marker development. She holds a habilitation in plant functional genomics at the University of Natural Resources and Life Sciences, Vienna, Austria. Her main research interests address genetic diversity and plant functional genomics.

## Abbreviations:

ACP: acyl carrier protein
AP: after pollination
CN: cetane number
EMS: ethyl methanesulfonate
EST: expressed sequence tag
FAD2: oleoylphosphatidylcholine Δ-12 desaturase
FA: fatty acid
GGPP: geranylgeranyl diphosphate
GGPPS: geranylgeranyl diphosphate synthase
GPPS: geranyl diphosphate synthase
KAS: beta-ketoacyl-ACP synthase
PE: phorbol ester
PKC: protein kinase C
QTL: quantitative trait locus
RIP: ribosome inactivating protein
SNP: single nucleotide polymorphism
TAG: triacylglycerol
